# Pain and physical activity levels among Rheumatoid Arthritis patients between the ages of 18 to 50 years in South Africa

**DOI:** 10.17159/2078-516X/2022/v34i1a11555

**Published:** 2022-01-01

**Authors:** RM Wilkinson, L Smith, S Ferreira

**Affiliations:** Department of Sport and Movement Studies in the Faculty of Health Sciences, University of Johannesburg, Doornfontein Campus, P.O. Box 524, Auckland Park, 2006, Johannesburg, South Africa

**Keywords:** inflammatory disease, exercise, physical limitations

## Abstract

**Background:**

Little epidemiological research on rheumatoid arthritis (RA) has been done in Africa, suggesting that it is an uncommon illness. In rural South Africa, RA has an overall prevalence of 0.07% and a prevalence of 2.5% in urban areas; therefore, it is not as uncommon as perceived by the lack of research. Patient-centred programmes to improve physical function have been lacking and, as a result, the prior assumption was that physical activity should be avoided.

**Objectives:**

To determine pain and physical activity levels among RA patients between the ages of 18 to 50 years in South Africa.

**Methods:**

A combination of two questionnaires were used, namely, the Global Physical Activity Questionnaire (2002) and the Pain Outcomes Questionnaire (2003). The collated questionnaires were distributed by rheumatologists and on social media platforms to RA patients between the ages of 18 to 50 years old living in South Africa. This study had a sample size of 105 participants, with participation occurring through the online Google forms platform.

**Results:**

One hundred and five participants with RA were recruited with an average age of 38±9 years. Most of the participants were females (93.3%). Seventy-two percent of the sample was classified as physically active, where work, leisure and travel activities were considered. No significant correlation between pain and physical activity was evident (r=0.10; p=0.311). Results showed significant correlations between pain and personal grooming (r=0.30; p=0.002), pain and ambulation (r=0.60; p=0.000), and pain and stair climbing (r=0.60; p=0.000).

**Conclusion:**

Physical activity has proven to have multiple benefits for those suffering with RA. In this South African sample of RA patients, the majority were classified as physically active, and pain did not affect the activity levels of the involved participants. This study opens further research questions regarding RA prevalence in South Africa, and the type and intensity of physical activity that would be beneficial for RA.

Arthritis is a musculoskeletal disorder which holds the potential of being disabling. ^[1]^ Arthritis affects people worldwide, with disability and functional limitations being characteristics of the disorder. ^[1]^ Rheumatoid arthritis is characterised by systemic inflammation, which can result in joint damage, disability and functional limitations. ^[1]^ Disease-modifying anti-rheumatic drugs, anti-inflammatories and analgesics are the types of medications commonly prescribed for the management of RA. ^[1]^ However, when the systemic inflammation in RA is poorly controlled and patients follow an unhealthy lifestyle, they are at risk for developing various comorbidities. ^[1, 2, 3]^ The incidence of cardiovascular events in those diagnosed with RA is estimated to be double that compared to the general population, with cardiovascular disease (CVD) typically developing at an earlier age in this population. ^[2]^ Obesity further increases the comorbidity risk in those with RA, with obesity and poor body composition related to RA, pharmacology and to physical inactivity. ^[1, 3]^

It has been suggested that RA patients are less active due to joint manifestations relating to the disease as well as other ‘general’ barriers, yet regular physical activity is an effective treatment and management tool for RA. ^[2, 3]^ Currently, exercise and the broad range of treatment options for RA are trumped by medication as the favoured modality; however, when considering longevity, interventions such as exercise become essential. ^[2, 4]^ Physical activity as therapeutic management has been proven to possess a range of benefits, including the improvement of general health and functional ability, as well as the reduction of associated disability. ^[2]^ Furthermore, physical activity is suitable for most individuals and can be used in conjunction with prescribed medications, which may allow for a reduced dosage while simultaneously benefitting general health status. ^[1, 2]^ However, various international research studies demonstrate that only a small percentage of RA patients are physically active. ^[1, 5, 6]^ Several countries have conducted research on physical activity participation among RA patients, but with the unpredictable and changing landscape in South Africa, as well as its economic development, there is a need to assess the current physical activity levels in a local context, and to identify barriers to physical activity participation. ^[1, 5, 6]^ Therefore, the purpose of this study was to determine pain and activity levels at work, while travelling or during leisure activities of RA patients in a South African context. The objectives of the study were: (1) determine whether most physical activity is completed during work, travel or recreation; (2) quantify the amount of time spent sedentary on a normal day; (3) determine the correlation between the pain, physical activity level, ambulation and personal grooming; (4) determine self-reported physical activity levels, overall energy levels, strength and endurance; and (5) determine patients’ perceptions on injury, risk and safety of exercise.

## Methods

### Study design

This study was cross-sectional in design and quantitative data were collected and analysed. To achieve the aim of the study, a combination of two questionnaires were utilised: the Global Physical Activity Questionnaire (GPAQ) and the Pain Outcomes Questionnaire (POQ). The collated questionnaires were made available on the Google Forms platform, allowing participants to access and complete them online.

### Selection and description of participants

Various rheumatologists were contacted to distribute the Google Forms link to the patients in their practices who met the inclusion criteria of this study. Additionally, the link was published to RA support groups and social media platforms by the researcher.

A sample of 105 participants complying with the inclusion criteria were recruited by means of purposive sampling. Participants were diverse in terms of backgrounds, provinces and treating rheumatologists.

#### Inclusion criteria

- Clinically diagnosed with RA.
- Between the ages of 18 and 50 years at the time of data collection. The minimum age of 18 years was established to allow the participant to consent independently to participation in the research study. The exclusion of individuals older than 50 years of age was determined due to the relationship between increased age and comorbidities, which could impact physical activity levels and the performance of daily activities.
- Residents in South Africa at the time of data collection.
- Male or female.
- Internet access to complete the questionnaire.

### Ethical considerations

All participants were informed about the purpose of the research by means of an information letter and were required to provide consent before data collection commenced. The study participants were aware that participation was voluntary and that withdrawal from the study could only take place before submission of the questionnaire. Every precaution was taken to protect the privacy of the participants and confidentiality of their personal information was ensured. This study was approved by the institutional Research Ethics Committee (REC-171-2019).

### Questionnaires

As mentioned, two combined questionnaires were used to gather subjective data relating to the pain and physical activity levels of the participants. Although the questionnaires included demographic questions, no identifying data was gathered and therefore, participation was anonymous. The questions in the GPAQ were centred on the participants’ activity levels during work, travel and leisure. The POQ was adapted by removing questions that were not relevant to the aims and objectives of this study. Therefore, the POQ was centred on the participants’ overall pain levels, as well as how pain affects their daily activities.

### Statistical analysis

The data collected were quantitative in nature. Statistical analysis was completed using the Statistical Package for Social Science (SPSS) version 26.0 and included percentages, means, standard deviations and correlations. The Kolmogorov-Smirnov test was used to assess the normality of the distribution of the data. To determine the correlations between the variables of interest, the Pearson product-moment correlation coefficient was computed because normality of the data was established. A calculation of statistical significance was done yielding 5% as the level of significance.

## Results

### Demographics

A total of 105 RA patients took part in the present study, with the demographic results demonstrated in Table 1. The mean age of the participants was 38±9, and only 7 participants were of the male gender. The mean age of RA diagnosis was 32 years. Eighteen of the 105 participants were not currently seeking treatment for their condition. Majority of the participants resided in the Gauteng province, with only 36 participants residing elsewhere.

### Global Physical Activity Questionnaire

Many participants had declared that they were physically active across more than one of the categories presented in Table 2. Based on the general physical activity guidelines, 29 participants (27.6%) were classified as physically inactive, while 76 participants (72.4%) were classified as physically active. ^[3]^

Both vigorous and moderate intensity activity was performed at work by 10 (9.5%) and 43 (41%) of the participants respectively. The mean time spent being physically active at work at a vigorous intensity was 229 minutes with a mean of four and a half days, while the mean amount of time spent doing moderate intensity activity at work was 175 minutes with a mean of four days.

**Fig. 1:**
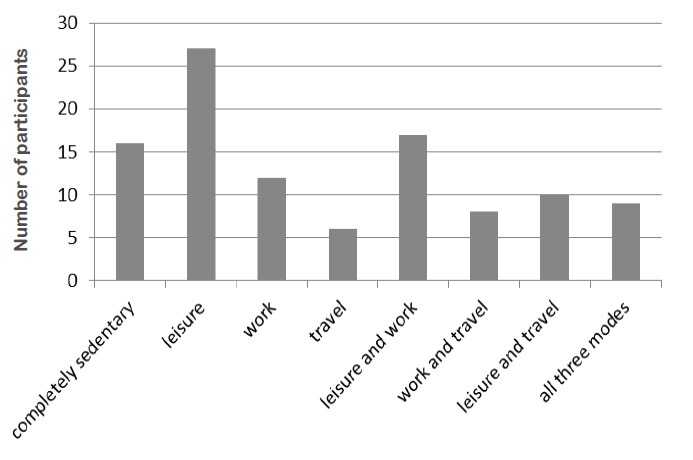
Categories of physical activity completed by the participants (n=105)

The number of participants in the different categories of physical activity is shown in Fig. 1. Sixteen participants (15%) performed no physical activity. Leisure activity had the highest number of participants and travel the least, with 27 (26%) and six (6%) respectively. Nine participants (9%) performed physical activity in all three categories.

The participants were required to select the amount of time they spend seated or reclining on a typical day. The categories of choice were 1–2 hours, 3–4 hours, 5–6 hours and >7 hours, with 12 (11.43%), 24 (22.86%), 42 (40%) and 27 (25.71%) selected respectively.

### Pain Outcomes Questionnaire

A 10-point Likert scale was used to respond to the POQ questions, results of which can be seen in Table 3. Out of 10, 5.8 was the mean pain level selected. The interference of their pain with daily activities yielded the following means: walking 5.3, carrying objects 5.9, stair climbing 5.5, and personal grooming 2.7. The participants’ mean rating of their personal physical activity, overall energy and strength and endurance was 4.9, 4.2 and 4.7 respectively. For depressive feelings on the day of the questionnaire, the mean response was 4.5. With regards to the fear of re-injuring themselves and exercise safety, the mean scores were 6.8 and 6.1 respectively, noting that none of the participants selected a score of zero for the last two questions.

### Categories of physical activities and associated mean pain level

In Table 4, when looking at the average pain levels reported for each of the categories of physical activity, the participants performing activity in both work and travel reported the highest mean pain levels (7.4±1.4 AU), followed by the participants performing no physical activity (6.4±2.3 AU). The lowest mean pain level reported was by the participants performing physical activity in the travel only category (3.7±2.8 AU).

### Correlations

Table 5 demonstrates the correlations of interest for the present study. The correlation between pain and total physical activity performed was *r*=0.10 for Pearson’s product moment correlations, demonstrating a slight effect of pain on physical activity participation. ^[7]^ The correlation between pain and the affected ability to walk was significant (*r*=0.60, *p=*0.0001) with a moderate positive correlation. ^[7]^ Another moderate positive correlation of *r*=0.60 was found between pain and the affected ability to climb stairs, also demonstrating a significance of p=0.0001. ^[7]^ Pain and the affected ability to manage personal grooming yielded a fair positive correlation of *r*=0.30, with significance of p=0.002. ^[7]^

## Discussion

The aim of this paper was to determine the relationships between pain and physical activity levels in a South African sample of RA patients between the ages of 18 and 50 years. During the study, the amount of physical activity performed during work, travel or leisure was determined and the amount of time patients are sedentary on a normal day was quantified. In addition, the correlation between pain, physical activity level, personal grooming, ambulation, and stair climbing was determined by means of a self-reported questionnaire. Two questionnaires were collated to achieve the above, namely the Global Physical Activity questionnaire and the Pain Outcomes questionnaire.

### Demographics

RA is said to affect 1% of the general population; therefore, it is expected that the sample size in this study would be smaller in comparison to studies centred around more prevalent conditions. ^[8]^ The prevalence of RA in South Africa ranges from 0.07% to 2.5% in rural and urban areas respectively. ^[9]^ The unequal distribution of gender in the study is consistent with numerous studies stating a higher prevalence among the female population. ^[1, 10]^ Rheumatoid arthritis has been shown to be four to five times more likely in females compared to males below the age of 50 years, but in African countries, the ratio is as large as 6:1 which is similar to that found in the sample in the present study. ^[10]^ The prevalence ratio tends to decrease in populations older than 60 years, where the female to male ratio is approximately 2:1. ^[10]^ In the present study, the mean age of diagnosis was 32 years, and 41.9% of the sample declared being diagnosed before 30 years of age. Research has reported the typical onset age for RA to be between 40 to 50 years; therefore, the mean of the present study is younger than this age. ^[1]^ A possible explanation for this is the age limitation for participation in the study, which was 18 to 50 years, and in addition, African samples have reported an average age of onset of 27 years. ^[11]^ The large distribution of participants located in the Gauteng province is attributed to Gauteng having the greatest population density, coupled with the highest internet connectivity in South Africa. ^[12]^

### Global Physical Activity Questionnaire (GPAQ)

When participants were asked about their participation in physical activity, results showed that 15.24% of the sample was sedentary and 72.4% were classified as physically active. Results also indicate that although only 15.24% of the sample declared that they were not partaking in any physical activity, a further 12.36% of the participants who were physically active were not performing sufficient levels of physical activity. The classification of sedentariness was determined in consultation with the American College of Sports Medicine (ACSM) guidelines on physical activity for psychological and physiological health benefits. ^[3]^ To be classed as physically active one must participate in 150 minutes of moderate intensity activity or 75 minutes of vigorous intensity activity, per week. ^[3]^ The results in the present study contrast with previous studies among American and European RA patients, which made use of standardised fitness tests to measure aerobic fitness, physical strength, agility, and endurance, as well as self-report questionnaires to determine physical activity participation, where 69% and 68% of participants, respectively, were classified as inactive. ^[1, 2]^ In addition, a study making use of the QUEST-RA (a quantitative clinical assessment of patients with rheumatoid arthritis seen in standard rheumatology care in 15 countries) was conducted on RA patients across 21 countries, which reported that only 13.8% of the participants were partaking in physical exercise three or more days per week. ^[6]^ There is a notable difference between these international studies and the present study, which could be attributed to the international studies collecting responses on exercise participation in isolation, in comparison to the present study which collected responses on physical activity during work, travel and leisure. ^[3]^ In 2020, a study conducted across 104 countries revealed that work, household, and travel activity measured with the GPAQ are important considerations when evaluating overall physical activity levels, especially in countries that continue to develop economically. ^[13]^ These are alternative forms of physical activity that are easily accessible and affordable to individuals who are susceptible to decreased physical activity due to societal and economic barriers. ^[13]^

Both vigorous and moderate intensity activity was performed by 37% of the participants in the present study. Although low impact and moderate intensity activity has been encouraged in this population for safety reasons, there is currently no direct evidence against vigorous intensity exercise participation in those with RA. ^[3]^ A pilot study among older RA patients reported that high-intensity interval training performed at levels exceeding that of the current health guidelines (of 150 minutes of moderate intensity activity or 75 minutes of vigorous intensity activity, per week, according to the ACSM) is associated with numerous benefits in this population. ^[14]^ Increased cardiorespiratory fitness and reduced disease activity were among many of the benefits experienced after ten weeks of vigorous intensity activity. ^[14]^ Evidence also exists that prolonged moderate to vigorous intensity activity reduces inflammatory markers, having a similar effect to medication on the treatment of RA, slows down disease progression, and reduces pain and fatigue. ^[14]^

In this study, 14.3% of the sample were unemployed. Due to the associated disability and cost incurred through treatment, RA affects patients economically. ^[4]^ Unemployment is a concern as it impacts the patient’s ability to afford the treatment aiding in the management of their condition. ^[4]^

Of those who participated, 65.71% spent five or more hours sitting or reclining on a typical day. Sedentary behaviour possesses serious health consequences by increasing the risk of both developing and progressing diseases while simultaneously affecting general health. ^[2, 15]^ In 13 international studies it was found that those who performed no physical activity and reclined for more than eight hours a day had a similar mortality risk to obese individuals and smokers. ^[15]^ It is important to note that prolonged sitting is a risk factor for all-cause mortality and may result in a viscous cycle between disease progression and health, therefore; it is essential for health interventions to minimise the amount of time spent seated. ^[2, 15]^

### Pain Outcomes Questionnaire (POQ)

The main complaint from RA patients is pain, which is a result of joint inflammation. ^[1, 10]^ The mean pain level of the sample in this study was 5.8/10, with 7/20 being the most commonly selected response. A heightened pain response is seen in those with RA, as pain is a major symptom of the condition and generally impacts the modality of treatment. ^[16]^ By making use of a 0-to-10-point scale to assess pain, this study only focused on the intensity of pain. Other aspects of pain also need to be considered to determine the total impact of pain on the individual. ^[16]^

Difficulty with daily tasks and reduced quality of life are frequently seen in those with chronic musculoskeletal conditions, which consequently catalyses institutionalisation, dependency, and increased healthcare needs. ^[4]^ The responses for this study demonstrated a mean score of 5.3/10, 5.9/10 and 5.5/10 for difficulty walking, carrying everyday objects and climbing stairs, respectively. Considering the young and middle ages of the participants in this sample, this is concerning as there are apparent issues with their ability to perform activities of daily living which may intensify with increased age and time should the disease progress. ^[1]^

Although physical activity offers therapeutic management for RA, there is a consistent concern that exercise participation can or will exacerbate symptoms and joint damage. ^[8, 17]^ Of the rheumatologists who participated in the Iverson et al study, 83% believed that exercise was an effective approach to managing symptoms of RA, yet none deemed themselves knowledgeable enough on exercise participation to correctly advise their patients. ^[17]^ When asked to rate their physical activity levels, the most commonly selected response by the participants was 5/10, which is logical considering that 72.4% of the sample were achieving the recommended amount of physical activity in a week. ^[3]^ Notably, only 19% of the sample selected a seven or more out of 10 for their physical activity. Physically active individuals appear to have a greater capability to overcome the associated barriers of participating in physical activity. ^[8]^ For patients who are not physically active, rheumatologists advocated a “mind shift” and proposed that positive mental health is achieved prior to the initiation of a physical activity programme. ^[17]^

The mean energy levels selected by the participants was 4.2/10, noting that no participant rated their energy level as 10/10. According to the literature, one of the reported symptoms of RA is mental and physical fatigue, hence this finding was not completely unexpected. ^[1, 8]^ The mean rating of experiencing symptoms of depression was 4.5/10, with the most commonly selected answer being four. In a 2019 study of RA patients, 55% of the participants reported having mild or worse symptoms of depression and 22% experienced moderate or worse symptoms of depression. ^[18]^ Of those participants, only 12% were seeking treatment for depression. ^[18]^

As mentioned above, the concern and fear of worsening the condition or causing injury when participating in physical activity is one of the most frequently mentioned barriers to physical activity participation in this population. ^[8, 17]^ This study enquired about the fear of re-injury with physical activity participation and the mean response was 6.8/10, with the most common response being eight. Regarding feelings around the safety of exercise for RA, 80% of the sample selected five or higher out of 10 (with 10 being ‘extremely safe’). Of the research studies that have been completed on the safety of physical activity and exercise participation in the RA population, little data has demonstrated that it negatively impacts on the condition. ^[2, 5, 8]^ Rather, extensive research has advocated for physical activity participation due to its benefits for RA patients. ^[2, 5, 8]^

### Categories of physical activity and mean pain levels

Additional analysis of the data examined the mean pain levels reported for the different physical activity categories. Looking at the different categories, the highest average pain level experienced was in the category of work and travel activity (7.4 AU). The mode and intensity of physical activity needs to suite the individual in terms of condition and abilities, as well as being ‘balanced’, with sufficient recovery time. Thus, one’s work-related physical activity demands may not be entirely appropriate in this regard. ^[4]^ The sedentary participants reported the second highest average pain level (6.4 AU), which is consistent with previous literature, as physical activity plays a role in reducing and aiding pain levels experienced in those with RA. ^[2, 8, 17]^ The travel only category (involving walking and/or cycling) had the lowest pain level, with an average of 3.7 AU. Active travel activities, such as walking and cycling, are seen in both high- and low-income countries, as beneficial for general health, and can contribute to one’s overall physical activity level. ^[13]^

### Correlations

An essential component of RA treatment is the management of pain experienced, with the prospect that effective pain management may promote compliance to therapy as well as fostering physical activity participation. ^[16]^ In the present study, it was established that pain had a slight positive effect on the physical activity participation in these participants. ^[7]^ Including travel, work and leisure activities could explain this unexpected result, as should one experience pain, participants may opt to avoid performing leisurely physical activity; however, should their work or travel require physical activity, they may have no option but to still participate in the activity to complete their tasks.

Chronic pain can substantially affect an individual’s ability to perform daily tasks. ^[19]^ The correlation between pain and affected ability to walk demonstrated that pain does impact the ability to walk through a moderate positive correlation. ^[7]^ Climbing stairs is another factor in ambulation, which also had a moderate positive correlation with pain. ^[7]^ The last correlation explored was between pain and the affected ability to manage personal grooming, which revealed a fair positive correlation. ^[7]^ All of these correlations were statistically significant. According to Edemekong et al, there are two categories of daily activities. The first category is associated with basic skills that are required to achieve and maintain basic needs, such as eating, grooming, and transferring from one position to another. ^[20]^ The second or instrumental category requires more skill, usually mentally and physically, such as transport, cleaning, and shopping. ^[20]^ This may explain why grooming is a lower rate disability as it falls within the basic category, can be completed in supine or seated positions, and generally requires less strength and mobility than other daily tasks such as walking and climbing stairs. ^[20]^ In a study completed in Spain on chronic disease patients, a disability for walking and climbing stairs existed, and when compared to other daily activities, personal grooming was one of the disabilities with a lower rating. ^[19]^

Although there has been further research on the treatment of RA, it remains a limiting, chronic disease which greatly impacts on the patient’s life, abilities, and morbidity. ^[4]^ In contrast to previously mentioned international studies, this study demonstrated that the majority of RA patients were labelled as physically active. ^[1, 5, 6]^ According to current literature, participation in physical activity and exercise is both beneficial and safe for these patients, with a wide range of psychological and physiological benefits. ^[2, 17]^ Some of the concerns surrounding physical activity in this population include the appropriate intensity, frequency and mode of activity, the possibility of causing further joint damage during activity, how joint pain will impact physical activity, and limited knowledge among healthcare professionals in terms of physical activity as therapeutic management. ^[8, 17]^ However, addressing these barriers and concerns will assist in encouraging and increasing activity levels in those with RA. ^[8, 17]^ Preserving one’s physical abilities and decreasing additional health risks are the main goals for physical activity in the RA population. ^[2]^ One does need to be aware of the unique barriers (both physical and mental), motivators and perceptions that may influence physical activity levels among RA patients. ^[8, 17]^

### Study limitations

The limitations in this study’s data collection methods include the distribution of the questionnaire using online platforms, resulting in the exclusion of RA patients without internet access. In addition, a small sample size was obtained. The omission of questions regarding the types of treatment sought was a limitation in data collection. By using the two selected questionnaires, only physical activity levels and pain outcomes were assessed. The authors acknowledge that additional questionnaires, such as a quality-of-life questionnaire, may have enhanced this study.

### Recommendations for future research

Performing a pre-screening assessment before the completion of the questionnaire may assist in excluding participants who have comorbidities that may negatively impact their responses. Including a question on the type of treatment that participants are seeking at the time of participation may also be beneficial, as it would allow for the analysis of and correlation between, pain levels and the different treatment modalities. Lastly, replacing the Pain Outcomes Questionnaire with the Rheumatoid Arthritis Pain Scale Questionnaire may provide more accurate results, as the latter questionnaire is specific for RA.

## Conclusion

In conclusion, this study demonstrated that in a South African sample, majority of RA patients were classified as physically active. Furthermore, and unexpectedly, no significant correlation was found between pain and physical activity levels. This study brings to light the importance of physical activity and exercise in the RA population, while also reminding the relevant healthcare professionals to consider the unique barriers and concerns of such participation among those with RA.

## Figures and Tables

**Table 1:** Demographic results of the sample (n=105)

	Frequency	Percentage (%)	Mean± SD
**Age (years)**			
18–29	22	20.9	38 ± 9
30–39	30	28.6	
40–50	53	50.5	

**Gender**			
Females	98	93.3	
Males	7	6.7	

**Age of diagnosis (years)**			
0–17	18	17.1	32 ± 11
18–29	26	24.8	
30–40	30	28.6	
40–50	31	29.5	

**Seeking treatment**			
Yes	87	82.9	
No	18	17.1	

**Province of residence**			
Gauteng	69	65.7	
KwaZulu- Natal	11	10.5	
Free State	4	3.8	
Limpopo	1	0.95	
Northern Cape	2	1.9	
Western Cape	15	14.3	
North West	2	1.9	
Mpumalanga	1	0.95	

**Table 2:** Global Physical Activity Questionnaire data (n=105)

Category	Response	Frequency	Percentage (%)	Number of days	Time (minutes/day)
Work – vigorous intensity	Yes	10	9.5	4.5 ± 1.3	229 ± 146
	No	72	68.6		
	Unemployed	15	14.3		
	Student	8	7.6		

Work - moderate intensity	Yes	43	41.0	4.0 ± 1.9	175 ± 138
	No	39	37.1		
	Not applicable	23	21.9		

Travel - walking or cycling	Yes	33	31.4	4.7 ± 2.0	111 ± 157
	No	72	68.6		

Leisure, sports and recreation - vigorous intensity	Yes	37	35.2	3.1 ± 1.4	60 ± 26
	No	68	64.8		

Leisure, sports and recreation - Moderate intensity	Yes	56	53.3	2.8 ± 1.4	57 ± 38
	No	49	46.7		

Data expressed as mean ± SD unless indicated otherwise.

**Table 3:** Pain Outcomes Questionnaire data (n=105)

Question	Mean ± SD	Max value selected	Min value selected
Average pain levels during the last week (0=no pain at all; 10=worst possible pain)	5.8 ± 2.3	10	0
Does your pain interfere with your ability to walk (0= not at all; 10= all the time)	5.3 ± 3.1	10	0
Does your pain interfere with your ability to carry/handle everyday objects such as bag of groceries or books (0= not at all; 10= all the time)	5.9 ± 2.8	10	0
Does your pain interfere with your ability to climb stairs (0= not at all; 10= all the time)	5.5 ± 3.4	10	0
Does your pain require you to use a walker, cane, wheelchair or other device (0= not at all; 10= all the time)	0.9 ± 2.2	10	0
Does your pain interfere with your ability to manage your personal grooming (combing hair, brushing teeth, etc) (0= not at all; 10= all the time)	2.7 ± 2.8	10	0
How would you rate your physical activity (0= significant limitations with basic activity; 10= can perform vigorous activity without limitations)	4.9 ± 1.9	10	0
How would you rate your overall energy (0= totally worn out; 10= most energy ever)	4.2 ± 1.9	9	1
How would you rate your strength and endurance today (0= very poor; 10= very high)	4.7 ± 2.0	10	1
How would you rate your feelings of depression today (0= not at all depressed; 10= extremely depressed)	4.5 ± 2.5	10	0
How much do you worry about re-injuring yourself if you are more active (0= not at all;10= all the time)	6.8 ± 2.8	10	1
How safe do you think it is for you to exercise (0= not safe at all; 10= extremely safe)	6.1 ± 2.1	10	1

**Table 4:** The different categories of physical activity and the associated average pain levels

Category of exercise participation	Participants (%)	Pain (AU)
Completely sedentary	15	6.4 ± 2.3
Leisure	26	5.2 ± 2.1
Work	11	6.3 ± 2.9
Travel	6	3.7 ± 2.8
Leisure and work	16	5.8 ± 2.5
Work and travel	8	7.4 ± 1.4
Leisure and travel	9	5.3 ± 1.8
Leisure, travel and work	9	6.3 ± 1.2

Data expressed as mean ± SD unless indicated otherwise. AU, arbitrary units

**Table 5:** Pearson product moment correlations between variables of interest

Variables	r	p-value
Pain level and total activity time by each participant	0.10	0.31
Pain level and the affected ability to walk	0.60	0.0001*
Pain level and the affected ability to climb stairs	0.60	0.0001*
Pain level and the affected ability to manage personal grooming	0.30	0.002*

* indicates significance (p<0.05).
